# Plasmodium falciparum PfEMP1 Modulates Monocyte/Macrophage Transcription Factor Activation and Cytokine and Chemokine Responses

**DOI:** 10.1128/IAI.00447-17

**Published:** 2017-12-19

**Authors:** Natália Guimarães Sampaio, Emily Marie Eriksson, Louis Schofield

**Affiliations:** a Population Health and Immunity Division, Walter and Eliza Hall Institute of Medical Research, Parkville, Victoria, Australia; b Department of Medical Biology, University of Melbourne, Parkville, Victoria, Australia; c Australian Institute of Tropical Health and Medicine, James Cook University, Townsville, Queensland, Australia; University of South Florida

**Keywords:** NF-κB, PfEMP1, Plasmodium falciparum, cytokines, immunosuppression, innate immunity, macrophages, malaria, monocytes, transcription factors

## Abstract

Immunity to Plasmodium falciparum malaria is slow to develop, and it is often asserted that malaria suppresses host immunity, although this is poorly understood and the molecular basis for such activity remains unknown. P. falciparum erythrocyte membrane protein 1 (PfEMP1) is a virulence factor that plays a key role in parasite-host interactions. We investigated the immunosuppressive effect of PfEMP1 on monocytes/macrophages, which are central to the antiparasitic innate response. RAW macrophages and human primary monocytes were stimulated with wild-type 3D7 or CS2 parasites or transgenic PfEMP1-null parasites. To study the immunomodulatory effect of PfEMP1, transcription factor activation and cytokine and chemokine responses were measured. The level of activation of NF-κB was significantly lower in macrophages stimulated with parasites that express PfEMP1 at the red blood cell surface membrane than in macrophages stimulated with PfEMP1-null parasites. Modulation of additional transcription factors, including CREB, also occurred, resulting in reduced immune gene expression and decreased tumor necrosis factor (TNF) and interleukin-10 (IL-10) release. Similarly, human monocytes released less IL-1β, IL-6, IL-10, monocyte chemoattractant protein 1 (MCP-1), macrophage inflammatory protein 1α (MIP-1α), MIP-1β, and TNF specifically in response to VAR2CSA PfEMP1-containing parasites than in response to PfEMP1-null parasites, suggesting that this immune regulation by PfEMP1 is important in naturally occurring infections. These results indicate that PfEMP1 is an immunomodulatory molecule that affects the activation of a range of transcription factors, dampening cytokine and chemokine responses. Therefore, these findings describe a potential molecular basis for immune suppression by P. falciparum.

## INTRODUCTION

Malaria caused by Plasmodium falciparum remains a significant global disease burden. During asexual blood-stage infection, when parasites invade red blood cells (RBCs), severe disease complications can occur as a result of site-specific parasite sequestration, the release of toxic by-products, and an unbalanced inflammatory response (1). The immune response to malaria is complex and implicated in both protection, involving parasite clearance and host survival (2, 3), and pathogenesis due to excess inflammation (4–6). Innate immune cells are essential for antimalarial immunity, and monocytes/macrophages play a central role in this response, consisting of parasite phagocytosis and the release of antimicrobials (e.g., reactive oxygen/nitrogen) and proinflammatory cytokines and chemokines (e.g., tumor necrosis factor [TNF], interleukin-1β [IL-1β], and IL-6) (5, 7, 8). Additionally, monocytes and dendritic cells (DCs) enhance the antiparasitic response from other cells, including γδ T cells and NK cells (9, 10).

Monocytes/macrophages detect parasites through pattern recognition receptors (PRRs) that bind pathogen-associated molecular features (11). In humans, P. falciparum is detected by the Toll-like receptors (TLRs) TLR2, TLR4, and TLR9 and the inflammasomes NACHT, LRR, PYD domain-containing protein 3 (NLRP3), and absent in melanoma 2 (AIM2) (12, 13). Recognition initiates a signaling cascade that activates numerous transcription factors (TFs), resulting in the release of inflammatory cytokines and the stimulation of pathogen destruction. Consequently, pathogens have developed mechanisms to evade immune detection by PRRs, including the expression of proteins that modulate or inhibit receptor activation or signaling (13). Notably, immunity to malaria develops slowly and is rapidly lost, and malaria is associated with higher rates of secondary infections (14), suggesting that it suppresses host immunity (15, 16). However, the underlying mechanism for this inhibition is ineffectively understood and remains an important question in the field.

P. falciparum erythrocyte membrane protein 1 (PfEMP1) is a parasite-derived transmembrane protein displayed on the infected RBC (iRBC) membrane and is important for malaria pathology and immune evasion. PfEMP1 is produced by the parasite and subsequently transported and inserted into the host plasma membrane through specific protein transport pathways, which require functional Maurer's clefts and parasite proteins such as skeletal binding protein 1 (SBP1) and PfEMP1 trafficking protein 1 (PTP1). Genetic ablation of these proteins results in the arrest of PfEMP1 trafficking and no PfEMP1 presentation at the host plasma membrane surface (17–19). PfEMP1 is encoded by up to 60 variants, has a high recombination rate (20), and is monoallelically expressed (21), providing efficient evasion of antibody detection (22). Furthermore, PfEMP1 mediates iRBC cytoadherence to host cells by binding surface molecules such as CD36, intercellular adhesion molecule 1 (ICAM-1), and chondroitin sulfate A (CSA) (23, 24). This avoids parasite destruction in the spleen but also contributes to severe disease such as cerebral and placental malaria, due to parasite sequestration in specific organs (25–27). Although PfEMP1 has been suggested to be immunostimulatory (28, 29), we have previously shown that PfEMP1 inhibits early gamma interferon (IFN-γ) release from peripheral blood mononuclear cells (PBMCs), suggesting that this protein has immunomodulatory functions (30). Furthermore, PfEMP1 has also been reported to inhibit DC maturation (31), although this was shown to be a dose-dependent *in vitro* effect (32). Nevertheless, cell-specific molecular events associated with PfEMP1 immune modulation have not been investigated.

As monocytes/macrophages are important early responders to malaria infection and interact directly with parasites, we investigated the effect of PfEMP1 on these cells. We found that PfEMP1 modulated the activation of immune-related transcription factors and that this resulted in reduced cytokine and chemokine responses from monocytes/macrophages that were specific for the expression of VAR2CSA PfEMP1. This indicates that the interaction of PfEMP1 with monocytes/macrophages can result in immunosuppressive effects.

## RESULTS

### Transgenic parasite strains CS2-SBP1-KO and CS2-PTP1-KO do not have PfEMP1 on the iRBC surface.

**FIG 1:**
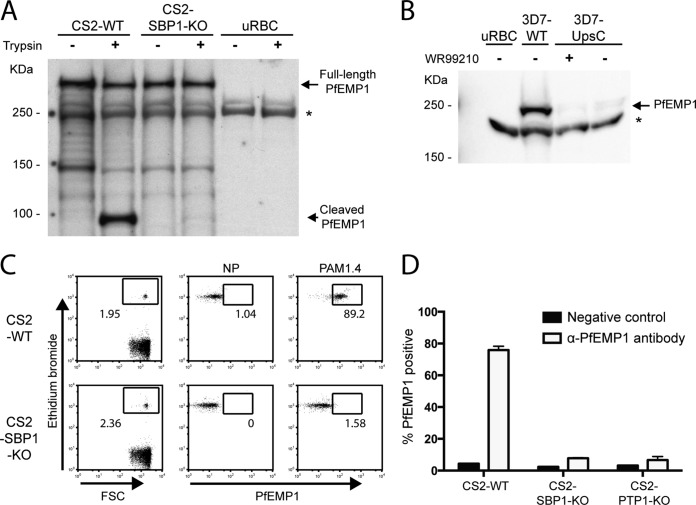
Transgenic parasite strains do not display PfEMP1 on the iRBC surface. (A) CS2-WT iRBCs, CS2-SBP1-KO iRBCs, or uRBCs were treated with 1 mg/ml trypsin or sham treated, and lysates were probed with an antibody against the cytoplasmic acidic terminal segment (ATS) of PfEMP1. Full-length VAR2CSA PfEMP1 is >300 kDa, and cleaved surface PfEMP1 is 90 kDa. The lack of the 90-kDa band represents the absence of PfEMP1 at the surface. Full-length 300-kDa PfEMP1 is still observed, as it represents the large intracellular pool of PfEMP1, which is protected from trypsin degradation. The asterisk indicates antibody cross-reactivity with the human spectrin protein. The Western blot is representative of results from three independent experiments. (B) 3D7-UpsC^R^ parasites were grown in the presence or absence of WR99210 for 2 weeks, and lysates from uRBCs, 3D7-WT iRBCs, and 3D7-UpsC^R^ iRBCs with or without WR99210 were probed with an anti-PfEMP1 ATS antibody. The Western blot is representative of results from three independent experiments. (C) Representative plots of CS2-WT and CS2-SBP1-KO cultures incubated with an antibody against the extracellular domain of VAR2CSA PfEMP1 (PAM1.4) and analyzed by flow cytometry, where ethidium bromide-positive cells were identified as iRBCs. NP, no primary antibody (used as a negative control); FSC, forward scatter. (D) Surface PfEMP1 expression levels of CS2-WT, CS2-SBP1-KO, or CS2-PTP1-KO were determined as the proportion of iRBCs that were PAM1.4 positive by flow cytometry. Error bars represent standard deviations of data from duplicates; the graph is representative of results from three independent experiments.

To study the potential immunomodulatory effects of PfEMP1 *in situ*, we used trophozoite-stage iRBCs from either PfEMP1-expressing strains (CS2 wild type and 3D7 wild type) or PfEMP1-null transgenic strains. CS2 parasites exclusively express one PfEMP1 variant (VAR2CSA) (24), whereas 3D7 parasites vary their PfEMP1 expression (20). The CS2-SBP1-KO strain is deficient in the export of PfEMP1 into the RBC cytoplasm and has no PfEMP1 on the surface but maintains normal trafficking and expression of other RBC membrane-associated proteins (17, 33). The loss of surface PfEMP1 was confirmed by iRBC trypsin treatment followed by Western blotting with an antibody specific to the cytoplasmic acidic terminal segment (ATS) of PfEMP1 (17–19). Trypsin degrades the extracellular domain of PfEMP1, but the intracellular ATS remains protected from trypsin degradation, resulting in a cleaved band on Western blots with CS2-WT (wild-type) iRBCs but not with CS2-SBP1-KO iRBCs (Fig. 1A). The surface PfEMP1 level was also measured by flow cytometry using an anti-PfEMP1 antibody (Fig. 1C) (34). Comparison of CS2 with CS2-SBP1-KO parasites and an additional PfEMP1 transport knockout (KO) strain (CS2-PTP1-KO) (18, 19) again demonstrated the absence of PfEMP1 on the surface of the KO strains (Fig. 1D). The 3D7-UpsC^R^ parasite strain contains the h*dhfr* gene that confers resistance to WR99210 downstream of the PfEMP1 promoter UpsC. Due to the allelic exclusivity of PfEMP1 expression, 3D7-UpsC^R^ does not express PfEMP1 in the presence of WR99210 (21). Western blot analysis of 3D7-WT and 3D7-UpsC^R^ lysates showed that PfEMP1 expression was inhibited in 3D7-UpsC^R^ iRBCs in the presence of WR99210 and that the removal of WR99210 allowed some recovery of PfEMP1 expression, but this remained substantially lower than that for 3D7-WT (Fig. 1B).

### PfEMP1 modulates specific transcription factor activation in macrophages.

**FIG 2:**
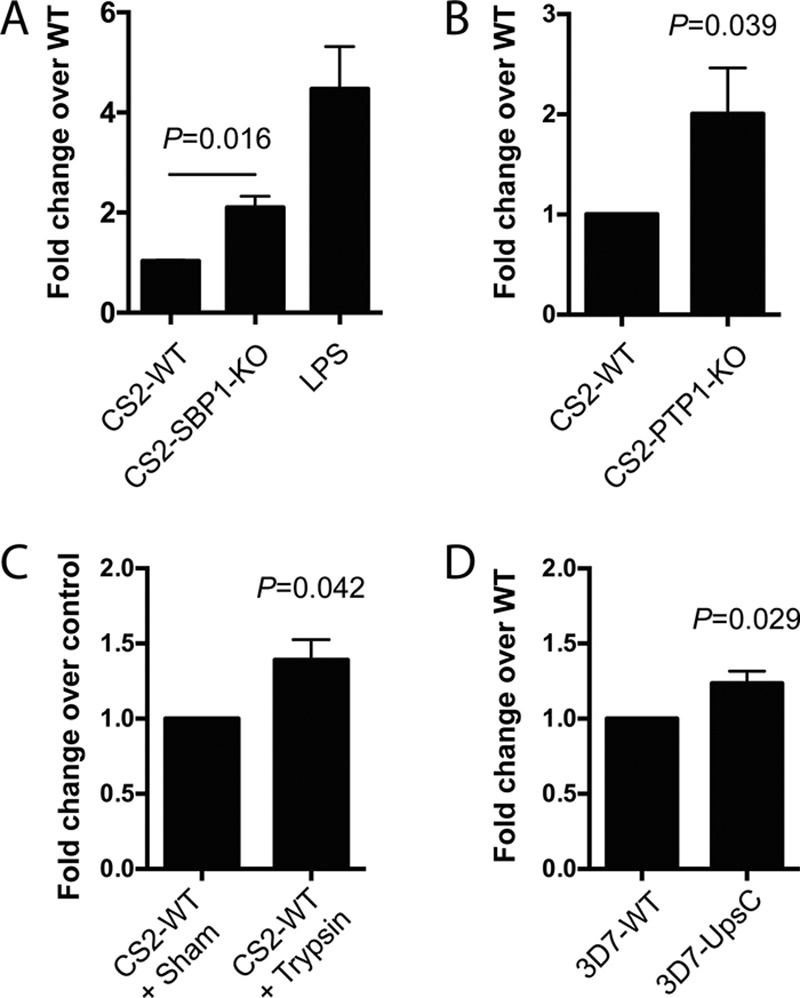
PfEMP1-null parasites induce greater NF-κB activation in macrophages than do wild-type parasites. RAW-ELAM cells were coincubated with uRBCs, CS2-WT iRBCs, CS2-SBP1-KO iRBCs, PBS (vehicle) control, or LPS (3.5 ng/ml; *n* = 6) (A); uRBCs or trypsin (1 mg/ml)- or sham-treated CS2-WT iRBCs (*n* = 4) (B); uRBC, CS2-WT iRBCs, or CS2-PTP1-KO iRBCs (*n* = 3) (C); and uRBCs, 3D7 iRBCs or 3D7-UpsC^R^ iRBCs (*n* = 4) (D). Luciferase activity was measured after 12 h, and uRBC background levels were subtracted from iRBC levels. Data were normalized as fold changes over WT values; the LPS level was normalized as the fold change over the value for the vehicle-only control. Data shown are means, and error bars represent standard errors of the means. Ratio-paired *t* tests were performed, and a *P* value of <0.05 was considered significant.

By using PfEMP1-null parasites, PfEMP1 was previously shown to downregulate IFN-γ production by PBMCs (30). As monocytes/macrophages are early responders to parasites during infection and major sources of cytokines and chemokines, we investigated potential effects of PfEMP1 on macrophage activation. To address this, we used RAW-ELAM macrophages, which have stable luciferase expression driven by the NF-κB-responsive ELAM (E-selectin) promoter (35) and are responsive to TLR activation (see Fig. S2 in the supplemental material). There was a significant increase in NF-κB activation in RAW-ELAM cells stimulated with the PfEMP1-null mutants CS2-SBP1-KO (*P* = 0.016) and CS2-PTP1-KO (*P* = 0.039) in iRBCs compared to that with CS2-WT (Fig. 2A and B). NF-κB activation was also increased when surface PfEMP1 was removed by trypsin treatment of wild-type CS2 (*P* = 0.042) (Fig. 2C). To ensure that the increase in NF-κB activation was due to the absence of PfEMP1 and not due to altered surface expression of other proteins, NF-κB activation was assessed by using the PfEMP1 knockout strain 3D7-UpsC^R^, which has varying PfEMP1 expression and is SBP1 and PTP1 competent (21). RAW-ELAM stimulation with 3D7-UpsC^R^ induced significantly higher NF-κB activation than that with 3D7-WT (*P* = 0.029) (Fig. 2D), although the magnitude of the difference was smaller than that for CS2 parasites. Collectively, the increased NF-κB activation by PfEMP1-null iRBCs indicates that the absence of cell surface PfEMP1 enhanced the activation of macrophages and that this was not exclusive to VAR2CSA PfEMP1.

**FIG 3:**
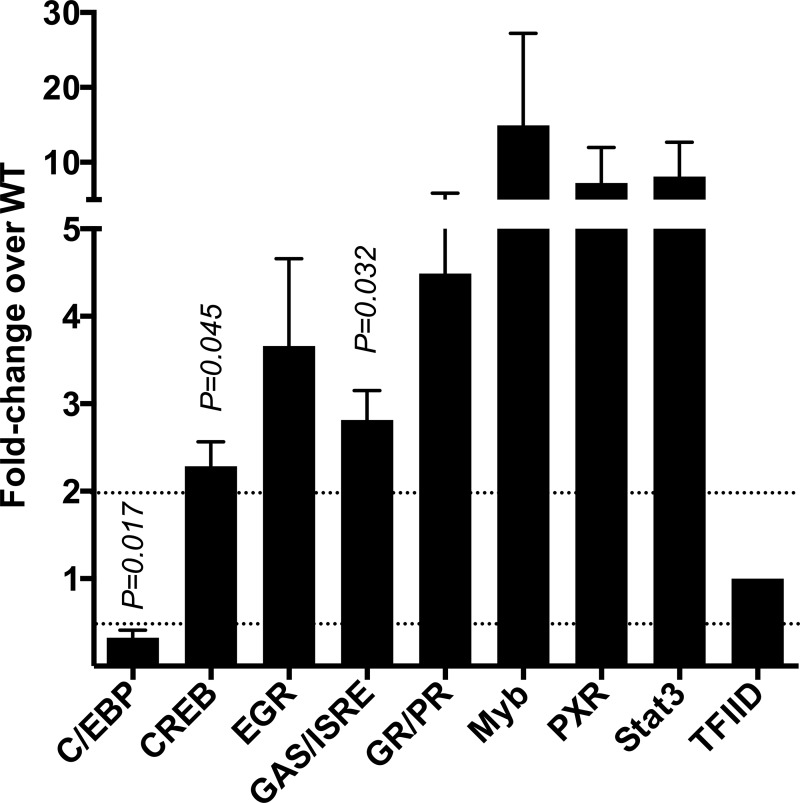
CS2-SBP1-KO parasites induce differential activation of a range of transcription factors in macrophages in comparison to CS2-WT parasites. Macrophages were coincubated with CS2-WT or CS2-SBP1-KO iRBCs for 4 h. Nuclear fractions were obtained, and activities of 48 transcription factors were measured by using a transcription factor activation profiling array (*n* = 3 independent experiments). Data were normalized to the value for the general transcription factor TFIID. Transcription factors found to have a minimum of a 2-fold change in three independent experiments are shown. Data shown are means, and error bars represent standard errors of the means. One-sample *t* tests were performed, and a *P* value of <0.05 was considered significant.

Since NF-κB activation was reduced in the presence of PfEMP1, we investigated the effect of PfEMP1 on other TFs. Subsequent experiments were performed with the CS2-WT and CS2-SBP1-KO parasite strains, as CS2 displays a consistent PfEMP1 phenotype that can easily be selected for high-level expression by panning on CSA (24). Macrophages were incubated with CS2-WT or CS2-SBP1-KO iRBCs for 4 h, and the activation of 48 different TFs was measured by using a TF activation profiling array. Of the 48 TFs tested, 6 consistently showed higher activity in response to CS2-SBP1-KO than in response to CS2-WT (CREB, EGR, GAS/ISRE, Myb, PXR, and Stat3) in three independent experimental repeats, of which the activities of CREB (2.3-fold; *P* = 0.045) and GAS/ISRE (2.8-fold; *P* = 0.032) were statistically significant (Fig. 3). Stimulation with CS2-SBP1-KO also showed a significant decrease in C/EBP-α activation compared to that with CS2-WT (0.3-fold; *P* = 0.017). These data demonstrate that PfEMP1 specifically modulates the activation of a range of TFs in macrophages.

### Parasites lacking surface PfEMP1 induce differential cytokine and chemokine responses in macrophages compared to wild-type parasites.

**FIG 4:**
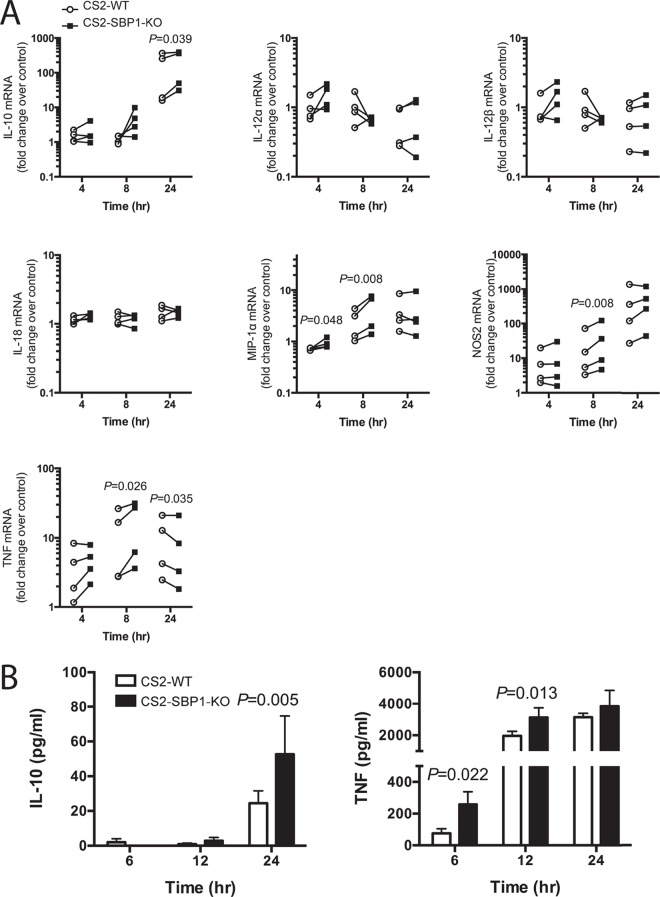
CS2-SBP1-KO parasites induce higher expression levels of immune response genes than do CS2-WT parasites. (A) Macrophages were stimulated with CS2-WT iRBCs (circles) or CS2-SBP1-KO iRBCs (squares) for the indicated times. RNA was extracted, and mRNA levels of TNF, IL-10, MIP-1α, NOS2, IL-12α, IL-12β, and IL-18 were measured by using RT-qPCR. Fold changes over the value for the no-stimulus control are shown (*n* = 4); ratio-paired *t* tests were performed, and a *P* value of <0.05 was considered significant. Connecting lines indicate paired CS2-WT- and CS2-SBP1-KO-stimulated samples from the same experimental repeat. (B) Macrophages were stimulated with uRBCs, CS2-WT iRBCs, or CS2-SBP1-KO iRBCs for indicated times; cell culture supernatants were collected; and levels of TNF and IL-10 were measured by an ELISA. Data shown are those after subtraction of the uRBC background (*n* = 3) and represent means; error bars represents standard errors of the means. Ratio-paired *t* tests were performed, and a *P* value of <0.05 was considered significant.

As the presence of PfEMP1 resulted in altered TF activation, we examined if this corresponded to a change in the expression of immunity-associated genes. The expression levels of the cytokines TNF, IL-10, IL-12α, IL-12β, and IL-18; the chemokine MIP-1α (CCL3); and the antimicrobial enzyme NOS2 (inducible nitric oxide synthase [iNOS]) were measured in macrophages after 4-, 8-, and 24-h stimulations with CS2-WT or CS2-SBP1-KO iRBCs. Compared to CS2-WT, CS2-SBP1-KO induced higher expression levels of TNF at 8 h (*P* = 0.026), MIP-1α at 4 and 8 h (*P* = 0.048 and *P* = 0.008), NOS2 at 8 h (*P* = 0.008), and IL-10 at 24 h (*P* = 0.039). CS2-SBP1-KO also induced lower expression levels of TNF at 24 h than those induced by CS2-WT (*P* = 0.035) (Fig. 4A), representing a shift in the kinetics of TNF induction, which was greater with CS2-SBP1-KO, followed by earlier downregulation. No induction of IL-12α, IL-12β, or IL-18 expression was observed when macrophages were stimulated with either CS2-WT or CS2-SBP1-KO iRBCs (Fig. 4A). At the protein level, significantly more TNF at 6 h (3.3-fold; *P* = 0.022) and 12 h (1.6-fold; *P* = 0.013) and IL-10 at 24 h (2.2-fold; *P* = 0.005) (Fig. 4B) were released in response to CS2-SBP1-KO than in response to CS2-WT.

### VAR2CSA PfEMP1 is associated with changes in cytokine and chemokine production from human monocytes.

**FIG 5:**
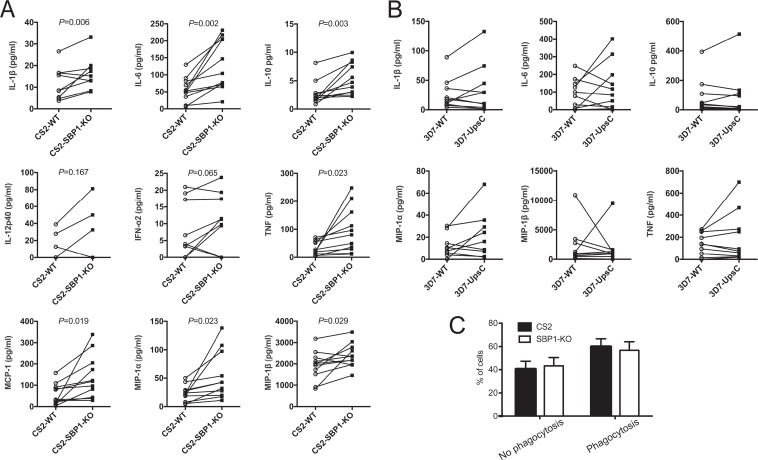
CS2-SBP1-KO parasites induce higher levels of cytokines and chemokines from primary human monocytes than do CS2-WT parasites. (A and B) Monocytes from naive human donors were stimulated for 12 h with uRBCs, CS2-WT iRBCs, and CS2-SBP1-KO iRBCs (*n* = 11 donors) (A) or uRBCs, 3D7-WT iRBCs, and 3D7-UpsC^R^ iRBCs (*n* = 10 donors) (B). Concentrations of cytokines and chemokines in culture supernatants were measured by a multiplex ELISA. Data shown are those after subtraction of the uRBC background, and connecting lines indicate paired WT- and mutant iRBC-stimulated samples. Different donors were used for the data in panels A and B. Paired *t* tests were performed, with a *P* value of <0.05 being considered significant. (C) Cells from three donors stimulated as described above for panel A were lifted, attached to slides by cytospinning, fixed, and Field stained. Slides were analyzed by light microscopy, and cells showing phagocytosis of iRBCs or hemozoin were counted. A minimum of 300 cells was counted under each condition; cell numbers were normalized and are displayed as proportions of the total cells counted. Error bars represent standard errors of means. Paired *t* test showed no significant difference in phagocytosis rates.

To extend our study of the modulatory role of PfEMP1, we investigated cytokine responses from human primary monocytes. Monocytes negatively isolated from naive human blood samples (*n* = 11 donors) were cocultured with CS2-WT or CS2-SBP1-KO iRBCs for 12 h. Levels of IFN-α2, IL-1β, IL-6, IL-10, IL-12p40, MCP-1, MIP-1α, MIP-1β, and TNF in the supernatant were measured by using a Luminex multiplex enzyme-linked immunosorbent assay (ELISA). Consistent with the effect on murine macrophages, the magnitude of the human monocyte response to CS2-SBP1-KO iRBCs was greater than that of the response to CS2-WT (Fig. 5A). CS2-SBP1-KO iRBCs induced significantly higher levels of IL-1β (*P* = 0.006), IL-6 (*P* = 0.002), IL-10 (*P* = 0.03), MCP-1 (*P* = 0.019), MIP-1α (*P* = 0.023), MIP-1β (*P* = 0.029), and TNF (*P* = 0.023) than those induced by CS2-WT. These differences were not due to residual WR99210 used for the selection of CS2-SBP1-KO parasites, as the treatment of monocytes with WR99210 did not affect their cytokine response to the lipopolysaccharide (LPS) stimulus (see Fig. S3 in the supplemental material). No significant changes in IL-12p40 and IFN-α2 levels were observed between CS2-WT and CS2-SBP1-KO. IL-18 and IL-12p70 were undetectable (data not shown). Furthermore, there was no difference in monocyte phagocytosis of CS2-WT iRBCs compared to CS2-SBP1-KO iRBCs (Fig. 5C). To investigate if monocyte cytokine responses were also modulated with additional strains, monocyte cytokine responses to 3D7-WT and 3D7-UpsC^R^ iRBCs were examined (*n* = 10 donors). However, no significant differences in cytokine responses between the two strains (Fig. 5B) were observed.

### Pathways of the innate immune response and parasitic diseases are associated with the effect of PfEMP1 on monocytes/macrophages.

To gain insight into the specific pathway(s) affected by PfEMP1, a list of the biomarkers modulated by PfEMP1 was generated (see Table S2 in the supplemental material) and interrogated by using pathway overrepresentation analysis on the InnateDB website (http://www.innatedb.ca/) (36). The top five signaling pathways and diseases associated with these markers are shown in Table 1. Of note, PRR pathways, such as NOD-like receptor (NLR) and TLR pathways, were among the top five pathways. Moreover, parasitic infections, including malaria, were the most overrepresented diseases associated with the list, demonstrating the specificity of the analysis.

**TABLE 1:** Pathway overrepresentation analysis of PfEMP1-modulated biomarkers interrogated against the Kyoto Encyclopedia of Genes and Genomes database

Highly associated pathway	Highly associated disease
Cytokine-cytokine receptor interaction	American trypanosomiasis
NOD-like receptor signaling	Malaria
Toll-like receptor signaling	Amoebiasis
Intestinal immunity (IgA production)	African trypanosomiasis
Cytosolic DNA sensing	Leishmaniasis

## DISCUSSION

Monocytes and macrophages are crucial to the innate immune response to P. falciparum and directly interact with iRBCs during infection. In human malaria, monocytes are a major source of proinflammatory cytokines (5, 8, 37), and together with their phagocytic and antigen-presenting capabilities, they play an important role in the stimulation of the adaptive immune response (7). PfEMP1 is central to the host-pathogen interaction and was previously shown to downregulate early IFN-γ responses to iRBCs (30), although the mechanism of this effect was not investigated. The present study used complementary strategies to investigate the effect of PfEMP1 on both macrophages and primary human monocytes. In contrast to previous studies that used bacterially expressed PfEMP1 peptides at high molecular concentrations (28, 29), we compared wild-type parasites with transgenic parasites deficient in either PfEMP1 expression or iRBC surface display. Thus, we retained PfEMP1 in its native conformation and at biologically relevant concentrations. Although it is possible that the trafficking of proteins other than PfEMP1 is affected in the CS2-SBP1-KO strain, to date, disruption of SBP1 has been shown to affect only the iRBC surface expression of PfEMP1, with no effect on the other major parasite-derived surface proteins, KAHRP, RIFIN, STEVOR, and PfEMP3 (17, 33, 38). Therefore, this suggests that the effects observed in this study are PfEMP1 specific and not due to perturbations in other parasite proteins.

CS2 parasites exclusively express VAR2CSA PfEMP1, which binds to CSA and is implicated in pregnancy-associated malaria, whereas 3D7 can vary its PfEMP1 expression (24, 26). Different PfEMP1 variants are associated with disease severity and clinical complications, due to differential binding to host proteins (26, 39–43). Our observations align with those of previous reports, as we discerned different downstream effects between the two parental strains in the present study. The PfEMP1-expressing CS2-WT and 3D7-WT strains both dampened the NF-κB response compared to the corresponding PfEMP1-deficient parasites, albeit the magnitude of the effect was less distinct with 3D7-WT. Furthermore, only the CS2 strain significantly affected monocyte cytokine release. Measurement of monocyte responses following stimulation with the 3D7 strains resulted in high variation in cytokine responses. In contrast to the phenotypically stable strain CS2, the relatively widespread distribution of responses observed with 3D7 may have been due to the altered PfEMP1 phenotype in cultured 3D7 parasites in combination with the inherent variability of human donors. While the PfEMP1-mediated inhibitory effect observed here may be exclusive to VAR2CSA PfEMP1, the increased NF-κB activation with 3D7-UpsC^R^ compared to that with 3D7-WT suggests that other PfEMP1 variants in addition to VAR2CSA result in significant effects on TF modulation. Further work is required to determine the degree to which monocyte activation is modulated by specific PfEMP1s and how this might be linked to cytoadhesion phenotypes.

VAR2CSA PfEMP1 on the iRBC surface was associated with the decreased activation of NF-κB-, CREB-, and GAS/ISRE-binding factors in macrophages and, consequently, with reduced immunity-associated gene expression and cytokine release. These findings demonstrated that the effect of PfEMP1 on TF activation translated to diminished immune responses to iRBCs. This suggests that although relatively small changes were observed in some cases, these signals were sufficient to distinguish meaningful effects on target gene expression. NF-κB-, CREB-, and GAS/ISRE-binding factors are activated by proinflammatory stimuli and upregulate the expression of immunity-associated genes (44). Activated NF-κB and CREB induce the transcription of numerous cytokines (e.g., IL-6 and TNF), and NF-κB also upregulates the expression of chemokines (e.g., MCP-1) and adhesion molecules (e.g., ICAM-1 and ELAM) (45). GAS/ISRE-binding factors include the interferon regulatory factor (IRF) and signal transducer and activator of transcription (STAT) families, which promote the expression of NOS2, IL-6, and TNF, as well as type I interferons (46, 47). Although we could not specifically differentiate which GAS/ISRE-binding factors were affected by VAR2CSA PfEMP1, these factors are known mediators of immune responses (48, 49).

We also observed lower C/EBP-α activation by PfEMP1-null CS2-SBP1-KO parasites than by wild-type parasites, suggesting that VAR2CSA PfEMP1 increases C/EBP-α activation. This TF is highly expressed in the myeloid lineage and is necessary for macrophage differentiation (50). Although a previous report showed that C/EBP-α KO macrophages had a blunted cytokine response to LPS, no studies have investigated C/EBP-α activation in response to microbes (50). Additionally, upon immune stimuli, the downregulation of C/EBP-α expression occurs concomitantly with the upregulation of C/EBP-β, which is also important for cytokine induction (51). This balance may be crucial for the final cytokine output, and thus, the lower C/EBP-α activation by PfEMP1-null iRBCs than by the wild type could reflect a more activated/polarized macrophage phenotype.

Monocytes are important sources of cytokines and chemokines in human malaria infection (5, 8). We found that the inhibitory effect of VAR2CSA PfEMP1 observed in macrophages also translated to human primary monocytes, with less IL-1β, IL-6, IL-10, MCP-1, MIP-1α, MIP-1β, and TNF being released in response to wild-type iRBCs than in response to PfEMP1-null iRBCs. Since IL-12 and IL-18 levels are increased in malaria-infected individuals (8, 52) and promote IFN-γ release from NK and T cells (53, 54), we investigated whether PfEMP1 modulated IL-12 and IL-18 release. Bioactive IL-12 (IL-12p70) is composed of p40 and p35 subunits, and IL-12p40 is produced in excess compared to IL-12p70 (54). We detected IL-12p40 in response to iRBCs but in only 4 of 11 donors. Conversely, no IL-12p70 or IL-18 was detected following iRBC stimulation, despite monocytes being reported to be the source of these cytokines (8). However, released IL-12 can be difficult to detect *in vitro* following iRBC stimulation (8), and the lack of detectable IL-12 and IL-18 may be due to an absence of accessory activating signals, which were not provided in a purified monocyte/macrophage culture but might be required for a complete immune response (53). Similarly, the induction of mRNA expression may also require accessory cells, where IL-18 mRNA expression in RAW cells requires exogenous IFN-γ (55). While it appears that IL-12 and IL-18 were not induced in monocytes/macrophages by iRBCs *in vitro*, PfEMP1 may still affect these cytokines *in vivo*.

We have shown for the first time that the activation of immunoregulatory TFs such as NF-κB is dampened in the presence of PfEMP1, resulting in reduced proinflammatory responses with VAR2CSA in particular. While specific PfEMP1 variants, apart from VAR2CSA, were not investigated separately, these observations suggest that PfEMP1 has an inhibitory role during interactions with monocytes. Inhibition of NF-κB in the regulation of immune responses is also undertaken by other parasites, such as Leishmania donovani and Toxoplasma gondii (56, 57). Additionally, numerous pathogens inhibit host immune responses by modulating TLR signaling pathways (13). Since TLRs can detect Plasmodium ligands (11), and NF-κB is central to these pathways, PfEMP1 could act to inhibit TLR activation or downstream signaling. Furthermore, inflammasomes can detect hemozoin and associated DNA, releasing IL-1β in response (12). As IL-1β release was decreased by PfEMP1, these pathways might also be targets for PfEMP1-mediated modulation. In contrast, the release of IFN-α2 was not affected, indicating that the modulatory effect of PfEMP1 did not involve PRRs that upregulate type I interferons, such as the intracellular nucleic acid-sensing cGAS/STING pathway (12).

The proposition that PfEMP1 affects signaling from specific PRRs was supported by data from a bioinformatic analysis of the pathways associated with the effect of PfEMP1. This analysis showed that TLR and NLR signaling pathways were among the most highly associated pathways, suggesting that these pathways could represent targets for PfEMP1 modulation. The involvement of PRR pathways was anticipated, as these pathways have been shown to be engaged in the detection of Plasmodium antigens, and the cytokines affected are known to be upregulated downstream of these pathways (11–13). Although PfEMP1 is not a known ligand for PRRs, it can interact with other host surface receptors such as CD36 and ICAM-1 (23), and VAR2CSA in particular binds to CSA. As TLR2 activation requires CD36 (58), this represents an attractive candidate for mediating the modulatory effects of PfEMP1. However, CS2 parasites express only CSA-binding VAR2CSA PfEMP1 and do not bind CD36, indicating that the effect is not mediated through CD36. Furthermore, we did not observe differences between phagocytosis of wild-type and PfEMP1-null CS2 iRBCs, indicating that VAR2CSA PfEMP1-associated modulation did not result from an increased phagocytosed antigen load. Instead, differential cytokine production in the presence and absence of VAR2CSA PfEMP1 likely occurs through an interaction with an unknown surface molecule that modulates PRR ligand binding and/or downstream signaling. The precise molecular interactions driving this modulatory effect remain to be fully elucidated, and in-depth investigations of all host-interacting partners of PfEMP1, especially VAR2CSA, are needed. Furthermore, future studies to establish how different PfEMP1 variants affect innate immune cell responses, and potential links to disease severity, will be important to further advance our understanding of P. falciparum inhibition of immune responses during infection.

In this study, we established a novel aspect of PfEMP1 immunosuppression through modulations of TF activation and subsequent VAR2CSA-specific reductions of cytokine and chemokine responses from monocytes/macrophages. As there is currently a malaria vaccine strategy targeting PfEMP1 (59, 60), it is important that the various functions of this protein are thoroughly and adequately investigated. Therefore, further efforts to reveal the diverse roles of PfEMP1 during malaria immune responses are warranted.

## MATERIALS AND METHODS

### Cell and parasite culture.

Reagents were purchased from Sigma-Aldrich unless otherwise specified. RAW-ELAM cells stably transfected with Renilla luciferase downstream of the ELAM (E-selectin) NF-κB-driven promoter were maintained in DMEM (Dulbecco's modified Eagle medium; Gibco) with 4.5 g/liter d-glucose, 40 mM sodium bicarbonate, 100 U/ml penicillin, and 100 μg/ml streptomycin (Gibco) and 10% heat-inactivated fetal calf serum (FCS; Gibco) at 37°C with 5% CO_2_.

Parasites were cultured as described previously (30). CS2 parasites with the disrupted SBP-1 (PF3D7_0501300) and PTP-1 (PF3D7_0202200 and PFB0106c) genes, referred to as CS2-SBP1-KO and CS2-PTP1-KO, respectively, and the 3D7-UpsC^R^ PfEMP1 knockdown strain were kindly provided by Alan Cowman (Walter and Eliza Hall Institute) (17, 18, 21). All KO strains were cloned and cultured in the presence of 4 nM WR99210 to maintain selective pressure. All cultures were screened for mycoplasma contamination on a monthly basis. Supernatants taken from 2-day cultures were collected and tested for mycoplasma by using the MycoAlert Plus kit (Lonza) according to the manufacturer's instructions and ascertained to be mycoplasma free.

To minimize differences in stimulation due to the variability in parasite maturation, cultures were synchronized to a 2-h window by 5% sorbitol treatment as described previously (61). Cultures were knob selected, and CS2 parasites were selected for binding to CSA (17).

For cell stimulations, trophozoite iRBCs (30 h postinvasion) were isolated by using a magnetically activated cell sorting (MACS) column (minimum of 95% purity; Miltenyi Biotec). Parasites were extensively washed with WR99210-free medium during isolation to ensure that no drug was present during coincubation experiments.

### Trypsin treatment of parasites.

iRBCs or uninfected RBCs (uRBCs) were sham or trypsin treated as previously described for cell stimulations (30) and lysate preparations (17).

### SDS-PAGE and Western blotting.

Lysates were analyzed on 3 to 8% Tris-acetate gels (NuPAGE; Thermo Fisher Scientific) and transferred to nitrocellulose (Criterion; Bio-Rad) according to the manufacturer's instructions. Membranes were blocked with 5% bovine serum albumin (BSA) in 0.05% NP-40–Tris-buffered saline (TBSN) and probed with mouse anti-ATS PfEMP1 antibody (clone 1B/98-6H1-1) preabsorbed to uRBC ghosts (17) and horseradish peroxidase (HRP)-conjugated anti-mouse IgG antibody (Cell Signaling Technology) in 0.5% milk in TBSN. The anti-ATS PfEMP1 antibody has been shown to broadly detect PfEMP1 in various parasite strains (17–19, 61).

### Fluorescence-activated cell sorter staining for VAR2CSA PfEMP1 on iRBCs.

Trophozoite iRBCs were washed and resuspended in 0.1% casein in phosphate-buffered saline (PBS). All staining solutions were prepared in 0.1% casein–PBS and incubated for 30 min at room temperature, followed by two washes. Samples were sequentially stained with PAM1.4 human anti-PfEMP1 antibody (1:100) (34), biotinylated anti-human antibody and streptavidin-Alexa 633 (both at a 1:200 dilution; ThermoFisher Scientific), and ethidium bromide (1:1,000; Bio-Rad). Samples were analyzed on a FACSCalibur instrument (BD Biosciences). Negative-control cells were stained with all antibodies but the primary antibody and used to set gates. PfEMP1-expressing iRBCs were identified as ethidium bromide- and Alexa 633-positive cells. Samples were analyzed in duplicate.

### NF-κB luciferase assay.

RAW-ELAM (35) cells (1 × 10^5^ cells/well) in phenol red-free DMEM were added to white 96-well plates and incubated for 12 to 16 h. Medium was removed, and purified iRBCs or uRBCs (5 × 10^5^ cells/well) in 100 μl medium were added. Plates were incubated for a further 12 h. BriteLite Plus luciferase reagent (100 μl; PerkinElmer) was added to wells, and the wells were incubated for 3 min. Total luminescence was measured on a Chameleon V plate reader (Hidex). Samples were analyzed in quadruplicate, and the vehicle negative control and the Ultrapure LPS-EB (lipopolysaccharide from E. coli 0111:B4; InvivoGen) positive control were included. Values for uRBC samples were subtracted from those for iRBC samples. Data were normalized to represent fold changes of PfEMP1-null iRBC stimulation over WT iRBC stimulation and of LPS stimulation over PBS stimulation, where the value for the WT was equal to “1,” and the luciferase levels measured from PfEMP1-null cells were determined relative to the WT signal. Independent experiments were repeated a minimum of three times on separate days and with freshly isolated iRBCs.

### Transcription factor array.

RAW-ELAM cells (3 × 10^6^ cells/well) were added to 6-well plates and incubated for 12 to 16 h. CS2-WT or CS2-SBP1-KO iRBCs (15 × 10^6^ cells/well) were added and incubated for 4 h. Cells were washed to remove iRBCs, and nuclear extracts were prepared by using a nuclear extraction kit (Signosis) according to the manufacturer's instructions. The activation levels of 48 TFs were measured by using TF Activation Profiling Plate Array I (Signosis). Briefly, biotin-labeled probes encoding TF DNA-binding-site consensus sequences were incubated with 8 μg of nuclear extract. Active TFs bound their respective probes, and unbound probes were washed away. Subsequently, bound probes were eluted and hybridized to complementary sequences on a 96-well plate. Luminescence was measured on a Chameleon V plate reader. Values were normalized to the TFIID value, and the fold change of CS2-SBP1-KO over CS2-WT was calculated. A ≥2-fold change between CS2-WT and CS2-SBP1-KO was considered a difference. Independent experiments were repeated three times on separate days and with freshly isolated iRBCs.

### Reverse transcription-quantitative PCR (RT-qPCR).

RAW-ELAM cells (3 × 10^6^ cells/well) were stimulated with iRBCs (15 × 10^6^ cells/well). RNA was extracted by using an RNeasy kit (Qiagen) according to the manufacturer's instructions. RNA (3 μg) was converted to cDNA by using a Transcriptor First Strand cDNA kit (Roche) with oligo(dT) primers. Quantitative PCR (qPCR) was performed with 10 ng cDNA in duplicate 12-μl reaction mixtures using FastStart Universal SYBR green Master (Roche), with 45 cycles and an annealing temperature of 60°C, on a LightCycler480 instrument (Applied Biosystems). Primers are described in Table S1 in the supplemental material. Data were processed by using LinRegPCR (62). Expression levels were normalized to glyceraldehyde-3-phosphate dehydrogenase (GAPDH) and β-actin values, and the fold change over unstimulated controls was calculated (63). Independent experiments were repeated four times on separate days and with freshly isolated iRBCs.

### Ethics statement.

Healthy adult human donors were recruited anonymously through the Volunteer Blood Donor Registry (approved by the human ethics committee at the Walter and Eliza Hall Institute, project no. 13/06). All subjects provided informed written consent.

### Monocyte isolation from whole blood.

Exclusion criteria for study subjects (40% male; median age, 45 years [range, 24 to 65 years]) were previously known malaria infection, recent severe illness, or recent travel to areas where malaria is endemic. PBMCs were isolated from whole blood (K_2_EDTA Vacutainer tubes; BD) within 2 h of collection, as described previously (30). Monocytes were enriched by using an EasySep Monocyte kit (Stem Cell Technologies) according to the manufacturer's instructions. Monocyte purity was verified by CD14 surface staining (CD14-fluorescein isothiocyanate [FITC] M5E2; BD) and analyzed on a FACSCalibur instrument. Purity was ∼80% on average (range, 75 to 95%) (see Fig. S1 in the supplemental material).

### Monocyte stimulation and cytokine ELISA.

Human monocytes (2 × 10^5^ cells) and purified iRBCs or uRBCs (6 × 10^5^ cells) were cocultured for 12 h as described previously (30). RAW-ELAM macrophages were stimulated with iRBCs or uRBCs as described above for the NF-κB luciferase assay. LPS (3.5 ng/ml), a positive control, and an unstimulated control were always included. Cell culture supernatants from triplicates were pooled and tested in duplicate. A multiplex ELISA (Bio-Plex Pro assay; Bio-Rad) on a Luminex platform and single ELISAs (ELISAkit) were done according to the manufacturers' instructions. For the phagocytosis assay, monocytes stimulated as described above were lifted by treatment with cold 10 mM glucose–3 mM EDTA in PBS for 10 min, attached to glass slides by cytospinning, methanol fixed, and Field stained (Quick-Dip; Fronine). Slides were analyzed by light microscopy (magnification, ×100), and cells showing no phagocytosis or phagocytosis of iRBCs or hemozoin were counted. A minimum of 300 cells was counted under each condition.

### Pathway overrepresentation analysis.

Identifiers obtained from the Ensembl database (http://www.ensembl.org/) for human and mouse genes and gene families (see Table S2 in the supplemental material) were uploaded to InnateDB (http://www.innatedb.ca/) for pathway overrepresentation analysis (36). The Kyoto Encyclopedia of Genes and Genomes (KEGG) database (http://www.genome.jp/kegg/pathway.html) was the source of annotated pathways. A hypergeometric algorithm and Benjamini-Hochberg correction were utilized automatically in the analysis to infer pathways or diseases with which the genes in the list are known to be significantly associated.

### Statistical analysis.

Data were analyzed by using GraphPad Prism 6. D'Agostino-Pearson normality tests were done for all data. For the NF-κB luciferase assay, RT-qPCR, and single-cytokine ELISAs, ratio-paired *t* tests were performed. For the TF activation array, one-sample *t* tests were performed, where samples were compared to a hypothetical value of 1. For Luminex multiplex ELISAs, parametric paired *t* tests were performed. *P* values of <0.05 were considered significant.

## Supplementary Material

Supplemental material

## References

[1] Schofield L, Grau GE 2005 Immunological processes in malaria pathogenesis. Nat Rev Immunol 5:722–735. doi:10.1038/nri1686. 16138104

[2] D'Ombrain MC, Robinson LJ, Stanisic DI, Taraika J, Bernard N, Michon P, Mueller I, Schofield L 2008 Association of early interferon-gamma production with immunity to clinical malaria: a longitudinal study among Papua New Guinean children. Clin Infect Dis 47:1380–1387. doi:10.1086/592971. 18947328

[3] Cabantous S, Poudiougou B, Traore A, Keita M, Cisse MB, Doumbo O, Dessein AJ, Marquet S 2005 Evidence that interferon-gamma plays a protective role during cerebral malaria. J Infect Dis 192:854–860. doi:10.1086/432484. 16088835

[4] Day NP, Hien TT, Schollaardt T, Loc PP, Chuong LV, Chau TT, Mai NT, Phu NH, Sinh DX, White NJ, Ho M 1999 The prognostic and pathophysiologic role of pro- and antiinflammatory cytokines in severe malaria. J Infect Dis 180:1288–1297. doi:10.1086/315016. 10479160

[5] Stanisic DI, Cutts J, Eriksson E, Fowkes FJI, Rosanas-Urgell A, Siba P, Laman M, Davis TME, Manning L, Mueller I, Schofield L 2014 γδ T cells and CD14^+^ monocytes are predominant cellular sources of cytokines and chemokines associated with severe malaria. J Infect Dis 210:295–305. doi:10.1093/infdis/jiu083. 24523513

[6] Awandare GA, Goka B, Boeuf P, Tetteh JKA, Kurtzhals JAL, Behr C, Akanmori BD 2006 Increased levels of inflammatory mediators in children with severe *Plasmodium falciparum* malaria with respiratory distress. J Infect Dis 194:1438–1446. doi:10.1086/508547. 17054074

[7] McGilvray ID, Serghides L, Kapus A, Rotstein OD, Kain KC 2000 Nonopsonic monocyte/macrophage phagocytosis of *Plasmodium falciparum*-parasitized erythrocytes: a role for CD36 in malarial clearance. Blood 96:3231–3240. 11050008

[8] Walther M, Woodruff J, Edele F, Jeffries D, Tongren JE, King E, Andrews L, Bejon P, Gilbert SC, De Souza JB, Sinden R, Hill AVS, Riley EM 2006 Innate immune responses to human malaria: heterogeneous cytokine responses to blood-stage *Plasmodium falciparum* correlate with parasitological and clinical outcomes. J Immunol 177:5736–5745. doi:10.4049/jimmunol.177.8.5736. 17015763

[9] Baratin M, Roetynck S, Pouvelle B, Lemmers C, Viebig NK, Johansson S, Bierling P, Scherf A, Gysin J, Vivier E, Ugolini S 2007 Dissection of the role of PfEMP1 and ICAM-1 in the sensing of *Plasmodium-falciparum*-infected erythrocytes by natural killer cells. PLoS One 2:e228. doi:10.1371/journal.pone.0000228. 17311092 PMC1794133

[10] Pichyangkul S, Saengkrai P, Yongvanitchit K, Stewart A, Heppner DG 1997 Activation of gammadelta T cells in malaria: interaction of cytokines and a schizont-associated *Plasmodium falciparum* antigen. J Infect Dis 176:233–241. doi:10.1086/514029. 9207372

[11] Eriksson EM, Sampaio NG, Schofield L 2013 Toll-like receptors and malaria—sensing and susceptibility. J Trop Dis 2:126–132.

[12] Gazzinelli RT, Kalantari P, Fitzgerald KA, Golenbock DT 2014 Innate sensing of malaria parasites. Nat Rev Immunol 14:744–757. doi:10.1038/nri3742. 25324127

[13] Hajishengallis G, Lambris JD 2011 Microbial manipulation of receptor crosstalk in innate immunity. Nat Rev Immunol 11:187–200. doi:10.1038/nri2918. 21350579 PMC3077082

[14] Scott JAG, Berkley JA, Mwangi I, Ochola L, Uyoga S, Macharia A, Ndila C, Lowe BS, Mwarumba S, Bauni E, Marsh K, Williams TN 2011 Relation between falciparum malaria and bacteraemia in Kenyan children: a population-based, case-control study and a longitudinal study. Lancet 378:1316–1323. doi:10.1016/S0140-6736(11)60888-X. 21903251 PMC3192903

[15] Greenwood BM, Bradley-Moore AM, Bryceson AD, Palit A 1972 Immunosuppression in children with malaria. Lancet i:169–172. doi:10.1016/S0140-6736(72)90569-7. 4109547

[16] Bejon P, Mwacharo J, Kai O, Todryk S, Keating S, Lowe B, Lang T, Mwangi TW, Gilbert SC, Peshu N, Marsh K, Hill AVS 2007 The induction and persistence of T cell IFN-gamma responses after vaccination or natural exposure is suppressed by *Plasmodium falciparum*. J Immunol 179:4193–4201. doi:10.4049/jimmunol.179.6.4193. 17785859 PMC2658805

[17] Maier AG, Rug M, O'Neill MT, Beeson JG, Marti M, Reeder J, Cowman AF 2007 Skeleton-binding protein 1 functions at the parasitophorous vacuole membrane to traffic PfEMP1 to the *Plasmodium falciparum*-infected erythrocyte surface. Blood 109:1289–1297. doi:10.1182/blood-2006-08-043364. 17023587 PMC1785152

[18] Maier AG, Rug M, O'Neill MT, Brown M, Chakravorty S, Szestak T, Chesson J, Wu Y, Hughes K, Coppel RL, Newbold C, Beeson JG, Craig A, Crabb BS, Cowman AF 2008 Exported proteins required for virulence and rigidity of *Plasmodium falciparum*-infected human erythrocytes. Cell 134:48–61. doi:10.1016/j.cell.2008.04.051. 18614010 PMC2568870

[19] Rug M, Cyrklaff M, Mikkonen A, Lemgruber L, Kuelzer S, Sanchez CP, Thompson J, Hanssen E, O'Neill M, Langer C, Lanzer M, Frischknecht F, Maier AG, Cowman AF 2014 Export of virulence proteins by malaria-infected erythrocytes involves remodeling of host actin cytoskeleton. Blood 124:3459–3468. doi:10.1182/blood-2014-06-583054. 25139348 PMC4246041

[20] Claessens A, Hamilton WL, Kekre M, Otto TD, Faizullabhoy A, Rayner JC, Kwiatkowski D 2014 Generation of antigenic diversity in *Plasmodium falciparum* by structured rearrangement of *var* genes during mitosis. PLoS Genet 10:e1004812. doi:10.1371/journal.pgen.1004812. 25521112 PMC4270465

[21] Voss TS, Healer J, Marty AJ, Duffy MF, Thompson JK, Beeson JG, Reeder JC, Crabb BS, Cowman AF 2006 A var gene promoter controls allelic exclusion of virulence genes in Plasmodium falciparum malaria. Nature 439:1004–1008. 16382237 10.1038/nature04407

[22] Bull PC, Lowe BS, Kortok M, Molyneux CS, Newbold CI, Marsh K 1998 Parasite antigens on the infected red cell surface are targets for naturally acquired immunity to malaria. Nat Med 4:358–360. doi:10.1038/nm0398-358. 9500614 PMC3836255

[23] Baruch DI, Gormely JA, Ma C, Howard RJ, Pasloske BL 1996 *Plasmodium falciparum* erythrocyte membrane protein 1 is a parasitized erythrocyte receptor for adherence to CD36, thrombospondin, and intercellular adhesion molecule 1. Proc Natl Acad Sci U S A 93:3497–3502. doi:10.1073/pnas.93.8.3497. 8622965 PMC39638

[24] Reeder JC, Cowman AF, Davern KM, Beeson JG, Thompson JK, Rogerson SJ, Brown GV 1999 The adhesion of *Plasmodium falciparum*-infected erythrocytes to chondroitin sulfate A is mediated by *P. falciparum* erythrocyte membrane protein 1. Proc Natl Acad Sci U S A 96:5198–5202. doi:10.1073/pnas.96.9.5198. 10220443 PMC21841

[25] Tembo DL, Nyoni B, Murikoli RV, Mukaka M, Milner DA, Berriman M, Rogerson SJ, Taylor TE, Molyneux ME, Mandala WL, Craig AG, Montgomery J 2014 Differential PfEMP1 expression is associated with cerebral malaria pathology. PLoS Pathog 10:e1004537. doi:10.1371/journal.ppat.1004537. 25473835 PMC4256257

[26] Jensen ATR, Magistrado P, Sharp S, Joergensen L, Lavstsen T, Chiucchiuini A, Salanti A, Vestergaard LS, Lusingu JP, Hermsen R, Sauerwein R, Christensen J, Nielsen MA, Hviid L, Sutherland C, Staalsoe T, Theander TG 2004 *Plasmodium falciparum* associated with severe childhood malaria preferentially expresses PfEMP1 encoded by group A *var* genes. J Exp Med 199:1179–1190. doi:10.1084/jem.20040274. 15123742 PMC2211911

[27] Salanti A, Dahlbäck M, Turner L, Nielsen MA, Barfod L, Magistrado P, Jensen ATR, Lavstsen T, Ofori MF, Marsh K, Hviid L, Theander TG 2004 Evidence for the involvement of VAR2CSA in pregnancy-associated malaria. J Exp Med 200:1197–1203. doi:10.1084/jem.20041579. 15520249 PMC2211857

[28] Donati D, Mok B, Chêne A, Xu H, Thangarajh M, Glas R, Chen Q, Wahlgren M, Bejarano MT 2006 Increased B cell survival and preferential activation of the memory compartment by a malaria polyclonal B cell activator. J Immunol 177:3035–3044. doi:10.4049/jimmunol.177.5.3035. 16920940

[29] Ndungu FM, Sanni L, Urban B, Stephens R, Newbold CI, Marsh K, Langhorne J 2006 CD4 T cells from malaria-nonexposed individuals respond to the CD36-binding domain of *Plasmodium falciparum* erythrocyte membrane protein-1 via an MHC class II-TCR-independent pathway. J Immunol 176:5504–5512. doi:10.4049/jimmunol.176.9.5504. 16622019

[30] D'Ombrain MC, Voss TS, Maier AG, Pearce JA, Hansen DS, Cowman AF, Schofield L 2007 *Plasmodium falciparum* erythrocyte membrane protein-1 specifically suppresses early production of host interferon-γ. Cell Host Microbe 2:130–138. doi:10.1016/j.chom.2007.06.012. 18005727

[31] Urban BC, Ferguson DJ, Pain A, Willcox N, Plebanski M, Austyn JM, Roberts DJ 1999 *Plasmodium falciparum*-infected erythrocytes modulate the maturation of dendritic cells. Nature 400:73–77. doi:10.1038/21900. 10403251

[32] Elliott SR, Spurck TP, Dodin JM, Maier AG, Voss TS, Yosaatmadja F, Payne PD, McFadden GI, Cowman AF, Rogerson SJ, Schofield L, Brown GV 2007 Inhibition of dendritic cell maturation by malaria is dose dependent and does not require *Plasmodium falciparum* erythrocyte membrane protein 1. Infect Immun 75:3621–3632. doi:10.1128/IAI.00095-07. 17470539 PMC1932960

[33] Chan J-A, Howell KB, Langer C, Maier AG, Hasang W, Rogerson SJ, Petter M, Chesson J, Stanisic DI, Duffy MF, Cooke BM, Siba PM, Mueller I, Bull PC, Marsh K, Fowkes FJI, Beeson JG 2016 A single point in protein trafficking by *Plasmodium falciparum* determines the expression of major antigens on the surface of infected erythrocytes targeted by human antibodies. Cell Mol Life Sci 73:4141–4158. doi:10.1007/s00018-016-2267-1. 27193441 PMC5042999

[34] Barfod L, Bernasconi NL, Dahlbäck M, Jarrossay D, Andersen PH, Salanti A, Ofori MF, Turner L, Resende M, Nielsen MA, Theander TG, Sallusto F, Lanzavecchia A, Hviid L 2007 Human pregnancy-associated malaria-specific B cells target polymorphic, conformational epitopes in VAR2CSA. Mol Microbiol 63:335–347. doi:10.1111/j.1365-2958.2006.05503.x. 17176260 PMC2779471

[35] Hume DA, Underhill DM, Sweet MJ, Ozinsky AO, Liew FY, Aderem A 2001 Macrophages exposed continuously to lipopolysaccharide and other agonists that act via Toll-like receptors exhibit a sustained and additive activation state. BMC Immunol 2:11. doi:10.1186/1471-2172-2-11. 11686851 PMC58839

[36] Breuer K, Foroushani AK, Laird MR, Chen C, Sribnaia A, Lo R, Winsor GL, Hancock REW, Brinkman FSL, Lynn DJ 2013 InnateDB: systems biology of innate immunity and beyond—recent updates and continuing curation. Nucleic Acids Res 41:D1228–D1233. doi:10.1093/nar/gks1147. 23180781 PMC3531080

[37] Chua CLL, Brown G, Hamilton JA, Rogerson S, Boeuf P 2013 Monocytes and macrophages in malaria: protection or pathology? Trends Parasitol 29:26–34. doi:10.1016/j.pt.2012.10.002. 23142189

[38] Cooke BM, Buckingham DW, Glenister FK, Fernandez KM, Bannister LH, Marti M, Mohandas N, Coppel RL 2006 A Maurer's cleft-associated protein is essential for expression of the major malaria virulence antigen on the surface of infected red blood cells. J Cell Biol 172:899–908. doi:10.1083/jcb.200509122. 16520384 PMC2063733

[39] Kaestli M, Cockburn IA, Cortés A, Baea K, Rowe JA, Beck H-P 2006 Virulence of malaria is associated with differential expression of *Plasmodium falciparum var* gene subgroups in a case-control study. J Infect Dis 193:1567–1574. doi:10.1086/503776. 16652286 PMC2877257

[40] Kyriacou HM, Stone GN, Challis RJ, Raza A, Lyke KE, Thera MA, Koné AK, Doumbo OK, Plowe CV, Rowe JA 2006 Differential *var* gene transcription in *Plasmodium falciparum* isolates from patients with cerebral malaria compared to hyperparasitaemia. Mol Biochem Parasitol 150:211–218. doi:10.1016/j.molbiopara.2006.08.005. 16996149 PMC2176080

[41] Rottmann M, Lavstsen T, Mugasa JP, Kaestli M, Jensen ATR, Müller D, Theander T, Beck H-P 2006 Differential expression of *var* gene groups is associated with morbidity caused by *Plasmodium falciparum* infection in Tanzanian children. Infect Immun 74:3904–3911. doi:10.1128/IAI.02073-05. 16790763 PMC1489729

[42] Cham GKK, Turner L, Lusingu J, Vestergaard L, Mmbando BP, Kurtis JD, Jensen ATR, Salanti A, Lavstsen T, Theander TG 2009 Sequential, ordered acquisition of antibodies to *Plasmodium falciparum* erythrocyte membrane protein 1 domains. J Immunol 183:3356–3363. doi:10.4049/jimmunol.0901331. 19675168

[43] Warimwe GM, Fegan G, Musyoki JN, Newton CRJC, Opiyo M, Githinji G, Andisi C, Menza F, Kitsao B, Marsh K, Bull PC 2012 Prognostic indicators of life-threatening malaria are associated with distinct parasite variant antigen profiles. Sci Transl Med 4:129ra45. doi:10.1126/scitranslmed.3003247. PMC3491874 22496547

[44] Kawai T, Sato S, Ishii KJ, Coban C, Hemmi H, Yamamoto M, Terai K, Matsuda M, Inoue J, Uematsu S, Takeuchi O, Akira S 2004 Interferon-α induction through Toll-like receptors involves a direct interaction of IRF7 with MyD88 and TRAF6. Nat Immunol 5:1061–1068. doi:10.1038/ni1118. 15361868

[45] Mayr B, Montminy M 2001 Transcriptional regulation by the phosphorylation-dependent factor CREB. Nat Rev Mol Cell Biol 2:599–609. doi:10.1038/35085068. 11483993

[46] Gao J, Morrison DC, Parmely TJ, Russell SW, Murphy WJ 1997 An interferon-gamma-activated site (GAS) is necessary for full expression of the mouse iNOS gene in response to interferon-gamma and lipopolysaccharide. J Biol Chem 272:1226–1230. doi:10.1074/jbc.272.2.1226. 8995425

[47] Takaoka A, Yanai H, Kondo S, Duncan G, Negishi H, Mizutani T, Kano S, Honda K, Ohba Y, Mak TW, Taniguchi T 2005 Integral role of IRF-5 in the gene induction programme activated by Toll-like receptors. Nat Cell Biol 434:243–249. 10.1038/nature03308 15665823

[48] Honda K, Taniguchi T 2006 IRFs: master regulators of signalling by Toll-like receptors and cytosolic pattern-recognition receptors. Nat Rev Immunol 6:644–658. doi:10.1038/nri1900. 16932750

[49] Li Z, Chao T-C, Chang K-Y, Lin N, Patil VS, Shimizu C, Head SR, Burns JC, Rana TM 2014 The long noncoding RNA THRIL regulates TNFα expression through its interaction with hnRNPL. Proc Natl Acad Sci U S A 111:1002–1007. doi:10.1073/pnas.1313768111. 24371310 PMC3903238

[50] Lee B, Qiao L, Lu M, Yoo HS, Cheung W, Mak R, Schaack J, Feng G-S, Chi N-W, Olefsky JM, Shao J 2014 C/EBPα regulates macrophage activation and systemic metabolism. Am J Physiol Endocrinol Metab 306:E1144–E1154. doi:10.1152/ajpendo.00002.2014. 24691027 PMC4025063

[51] Alam T, An MR, Papaconstantinou J 1992 Differential expression of three C/EBP isoforms in multiple tissues during the acute phase response. J Biol Chem 267:5021–5024. 1371993

[52] Malaguarnera L, Pignatelli S, Musumeci M, Simporè J, Musumeci S 2002 Plasma levels of interleukin-18 and interleukin-12 in *Plasmodium falciparum* malaria. Parasite Immunol 24:489–492. doi:10.1046/j.1365-3024.2002.00485.x. 12654091

[53] Newman KC, Korbel DS, Hafalla JC, Riley EM 2006 Cross-talk with myeloid accessory cells regulates human natural killer cell interferon-gamma responses to malaria. PLoS Pathog 2:e118. doi:10.1371/journal.ppat.0020118. 17154717 PMC1687207

[54] Trinchieri G 2003 Interleukin-12 and the regulation of innate resistance and adaptive immunity. Nat Rev Immunol 3:133–146. doi:10.1038/nri1001. 12563297

[55] Kim YM, Im JY, Han SH, Kang HS, Choi I 2000 IFN-gamma up-regulates IL-18 gene expression via IFN consensus sequence-binding protein and activator protein-1 elements in macrophages. J Immunol 165:3198–3205. doi:10.4049/jimmunol.165.6.3198. 10975835

[56] Srivastav S, Kar S, Chande AG, Mukhopadhyaya R, Das PK 2012 *Leishmania donovani* exploits host deubiquitinating enzyme A20, a negative regulator of TLR signaling, to subvert host immune response. J Immunol 189:924–934. doi:10.4049/jimmunol.1102845. 22685311

[57] Du J, An R, Chen L, Shen Y, Chen Y, Cheng L, Jiang Z, Zhang A, Yu L, Chu D, Shen Y, Luo Q, Chen H, Wan L, Li M, Xu X, Shen J 2014 *Toxoplasma gondii* virulence factor ROP18 inhibits the host NF-κB pathway by promoting p65 degradation. J Biol Chem 289:12578–12592. doi:10.1074/jbc.M113.544718. 24648522 PMC4007449

[58] Hoebe K, Georgel P, Rutschmann S, Du X, Mudd S, Crozat K, Sovath S, Shamel L, Hartung T, Zähringer U, Beutler B 2005 CD36 is a sensor of diacylglycerides. Nature 433:523–527. doi:10.1038/nature03253. 15690042

[59] Fried M, Duffy PE 2015 Designing a VAR2CSA-based vaccine to prevent placental malaria. Vaccine 33:7483–7488. doi:10.1016/j.vaccine.2015.10.011. 26469717 PMC5077158

[60] Gangnard S, Lewit-Bentley A, Dechavanne S, Srivastava A, Amirat F, Bentley GA, Gamain B 2015 Structure of the DBL3X-DBL4ε region of the VAR2CSA placental malaria vaccine candidate: insight into DBL domain interactions. Sci Rep 5:14868. doi:10.1038/srep14868. 26450557 PMC4598876

[61] McMillan PJ, Millet C, Batinovic S, Maiorca M, Hanssen E, Kenny S, Muhle RA, Melcher M, Fidock DA, Smith JD, Dixon MWA, Tilley L 2013 Spatial and temporal mapping of the PfEMP1 export pathway in *Plasmodium falciparum*. Cell Microbiol 15:1401–1418. doi:10.1111/cmi.12125. 23421990 PMC3711974

[62] Ruijter JM, Ramakers C, Hoogaars WMH, Karlen Y, Bakker O, van den Hoff MJB, Moorman AFM 2009 Amplification efficiency: linking baseline and bias in the analysis of quantitative PCR data. Nucleic Acids Res 37:e45. doi:10.1093/nar/gkp045. 19237396 PMC2665230

[63] Pfaffl MW 2001 A new mathematical model for relative quantification in real-time RT-PCR. Nucleic Acids Res 29:e45. doi:10.1093/nar/29.9.e45. 11328886 PMC55695

