# Supplementary material for: Plasmodium falciparum PfEMP1 Modulates Monocyte/Macrophage Transcription Factor Activation and Cytokine and Chemokine Responses
Source: Infect Immun. 2017 Dec 19;86(1):e00447-17. doi: 10.1128/IAI.00447-17 (PMC5736827; doi:10.1128/IAI.00447-17)
Supplement: Supplemental material [file supp_86_1_e00447-17__index.html]

Supplemental material 

# Plasmodium falciparum PfEMP1 Modulates Monocyte/Macrophage Transcription Factor Activation and Cytokine and Chemokine Responses

## Supplemental material

- Supplemental file 1 -

  Fig. S1. Negative selection of human monocytes from PBMC produced a highly enriched monocyte population. Fig. S2. TLR ligands induce NF-κB activation in RAW-ELAM cells in a dose-dependent manner. Fig. S3. Residual WR99210 does not affect monocyte cytokine responses. Table S1. qPCR primers for murine genes. Table S2. List of biomarkers modulated by PfEMP1, with respective ENSEMBL gene/family codes for mouse and human.

  PDF, 2.2M
