# Supplementary material for: Plasmodium falciparum PfEMP1 Modulates Monocyte/Macrophage Transcription Factor Activation and Cytokine and Chemokine Responses
Source: Infect Immun. 2017 Dec 19;86(1):e00447-17. doi: 10.1128/IAI.00447-17 (PMC5736827; doi:10.1128/IAI.00447-17)
Supplement: Supplemental material [file IAI.00447-17_zii001182247s1.pdf]

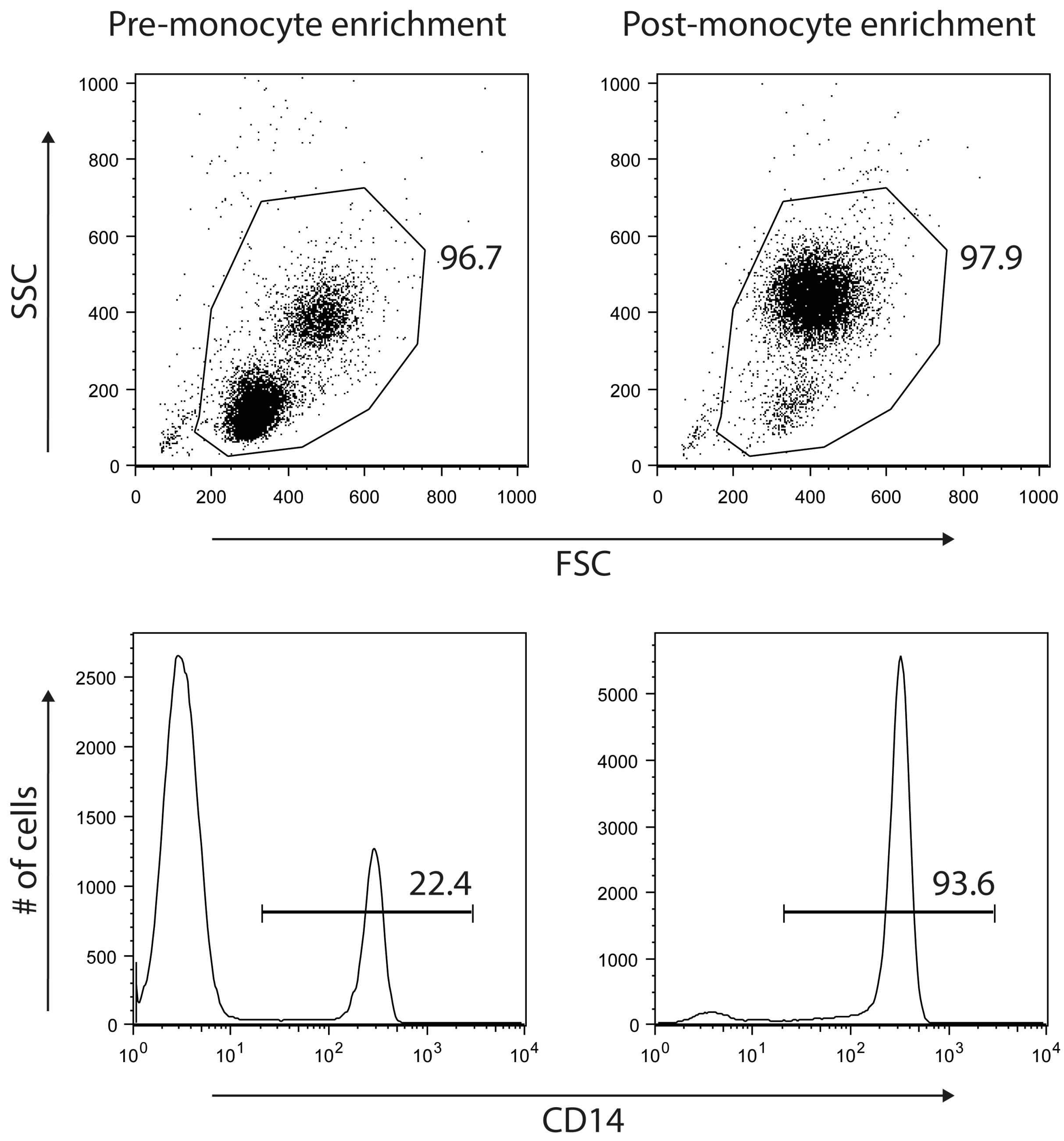

Supplementary Figure 1. Negative selection of human monocytes from PBMC produced a highly enriched monocyte population. 5 x 10^5 monocytes or PBMC were incubated with CD14-FITC antibody (clone M5E2, BD) on ice for 20 min. Cells were washed and fixed with 2% paraformaldehyde, and analyzed on a FACS Calibur. Representative plots for one donor are shown.

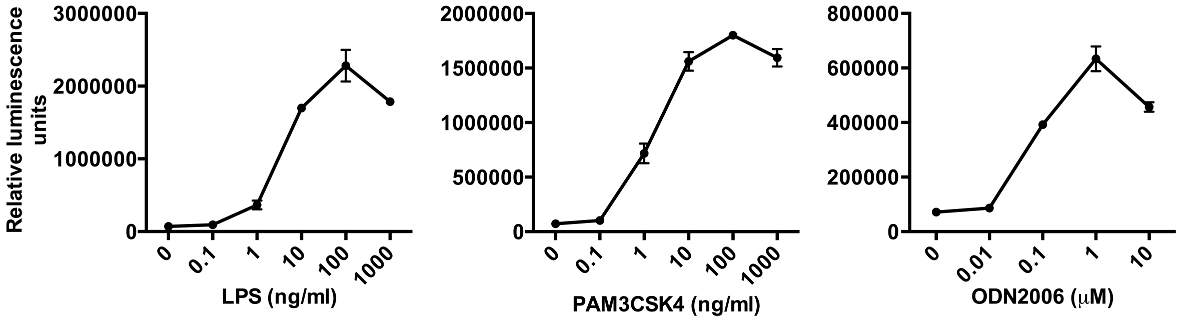

Supplementary Figure 2. TLR ligands induce NF-κB activation in RAW-ELAM cells in a dose-dependent manner. RAW-ELAM macrophages contain the NF-κB-binding ELAM promoter upstream of *Renilla luciferase,* and respond in a dose-dependent manner to TLR ligands LPS, PAM3CSK4 and ODN2006 after 12 hr stimulation.

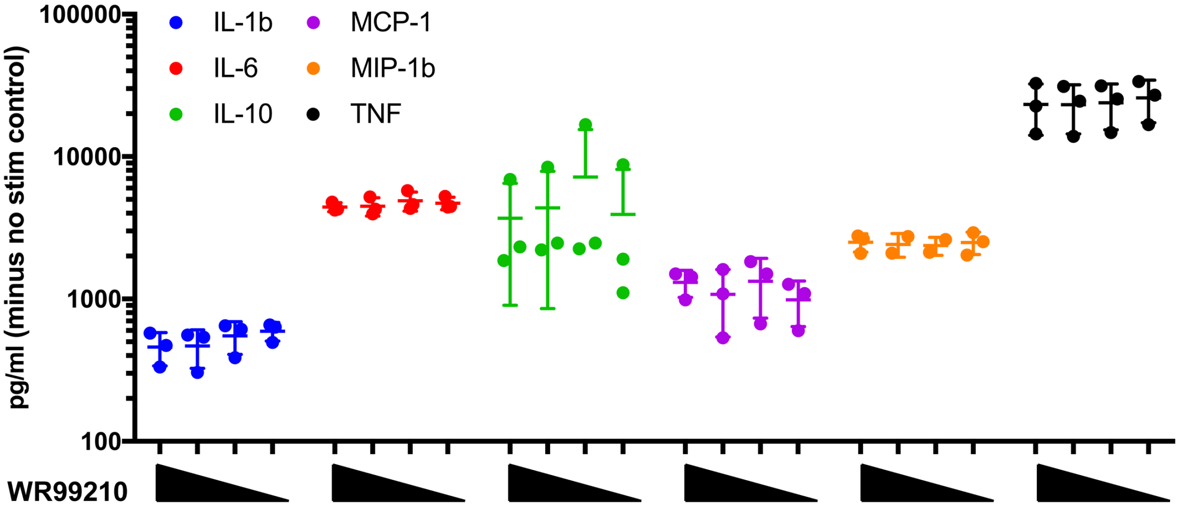

Supplementary Figure 3. Residual WR99210 does not affect monocyte cytokine responses. Monocytes (n = 3 donors) were stimulated for 12 hr with 5 ng/ml LPS or left unstimulated in presence of decreasing concentrations of WR (4, 0.4, and 0.04 pM) or media only. Cytokine levels in the supernatant were measured by Luminex multiplex ELISA. Cytokine levels for no stimulus control was subtracted from respective LPS stimulated conditions, and individual donor values are shown. Error bars represent standard deviation. Two-way ANOVA indicated no change in cytokine responses with WR99210 incubation, *P*=0.3758.

Supplementary Table 1. qPCR primers for murine genes.

Target mRNA	Forward primer sequence	Reverse primer sequence
TNF	5’-AGCCCACGTCGTAGCAAAC	5’-ACAAGGTACAACCCATCGGC
IL-10	5’- ATTTGAATTCCCTGGGTGAGAAG	5’- CACAGGGGAGAAATCGATGACA
MIP-1α (CCL3)	5’- CCAGGTGTCATTTTCCTGACT	5’- ATGCAGGTGGCAGGAATGTT
NOS2 (iNOS)	5’-GTTCTCAGCCCAACAATACAAGA	5’-GTGGACGGGTCGATGTCAC
GAPDH	5’- CTCCCACTCTTCCACCTTCG	5’- CCACCACCCTGTTGCTGTAG
β-actin	5’- AGAGGGAAATCGTGCGTGAC	5’- CAATAGTGATGACCTGGCCGT

Supplementary Table 2. List of biomarkers modulated by PfEMP1, with respective ENSEMBL gene/family codes for mouse and human.

Gene name	Human	Mouse
C/EBP-α	ENSG00000245848	ENSMUSG00000034957
CCL2 (MCP-1)	ENSG00000108691	ENSMUSG00000035385
CCL3 (MIP-1α)	ENSG00000277632	ENSMUSG00000000982
CCL4 (MIP-1β)	ENSG00000275302	ENSMUSG00000018930
CREB1	ENSG00000118260	ENSMUSG00000025958
IL-10	ENSG00000136634	ENSMUSG00000016529
IL-1β	ENSG00000125538	ENSMUSG00000027398
IL-6	ENSG00000136244	ENSMUSG00000025746
IRF family	ENSFM00500000271706	ENSFM00730001522091
NF-κB family	ENSFM00730001522091	ENSFM00730001522091
NOS2	ENSG00000007171	ENSMUSG00000020826
TNF	ENSG00000232810	ENSMUSG00000024401
